# Enhancing of Self-Referenced Continuous-Variable Quantum Key Distribution with Virtual Photon Subtraction

**DOI:** 10.3390/e20080578

**Published:** 2018-08-06

**Authors:** Hai Zhong, Yijun Wang, Xudong Wang, Qin Liao, Xiaodong Wu, Ying Guo

**Affiliations:** 1 School of Information Science and Engineering, Central South University, Changsha 410083, China; 2 School of IOT Engineering, Taihu University, Wuxi 214064, China

**Keywords:** quantum cryptography, continuous-variable quantum key distribution, photon subtraction

## Abstract

The scheme of the self-referenced continuous-variable quantum key distribution (SR CV-QKD) has been experimentally demonstrated. However, because of the finite dynamics of Alice’s amplitude modulator, there will be an extra excess noise that is proportional to the amplitude of the reference pulse, while the maximal transmission distance of this scheme is positively correlated with the amplitude of the reference pulse. Therefore, there is a trade-off between the maximal transmission distance and the amplitude of the reference pulse. In this paper, we propose the scheme of SR CV-QKD with virtual photon subtraction, which not only has no need for the use of a high intensity reference pulse to improve the maximal transmission distance, but also has no demand of adding complex physical operations to the original self-referenced scheme. Compared to the original scheme, our simulation results show that a considerable extension of the maximal transmission distance can be obtained when using a weak reference pulse, especially for one-photon subtraction. We also find that our scheme is sensible with the detector’s electronic noise at reception. A longer maximal transmission distance can be achieved for lower electronic noise. Moreover, our scheme has a better toleration of excess noise compared to the original self-referenced scheme, which implies the advantage of using virtual photon subtraction to increase the maximal tolerable excess noise for distant users. These results suggest that our scheme can make the SR CV-QKD from the laboratory possible for practical metropolitan area application.

## 1. Introduction

Quantum key distribution (QKD), which is the best-known application of quantum cryptography, is able to distribute a secret key between two distant legitimate parties, called Alice and Bob, over an a priori unsecure communication channel [1,2,3,4]. There are two branches in performing quantum key distribution: the discrete-variable (DV) QKD based on modulating a single photon state and the continuous-variable (CV) QKD based on coherent detection [5,6,7,8]. CV-QKD has demonstrated the advantages of high detection efficiency and low experiment cost. More significantly, most standard telecommunication technologies could be compatible with CV-QKD, which makes CV-QKD more attractive and hence fruitful [9,10,11,12,13,14].

The major research protocol of CV-QKD is the Gaussian modulated coherent state (GMCS) CV-QKD protocol, the unconditional security of which has been demonstrated in theory [15,16,17]. In order to provide a phase reference for Bob’s coherent detection on the received quantum signals, the conventional GMCS protocol needs to co-transmit a local oscillator (LO), a high bright classical beam, between Alice and Bob. However, due to the existence of the LO, a series of new, severe security loopholes has been proven, thus making some side-channel attacks possible [18,19,20,21,22], which can greatly reduce the overall security of the GMCS CV-QKD protocol. In order to obtain a more robust system against the aforementioned side-channel attacks, new schemes have been proposed in recent years [23,24,25]. These schemes waive the transmission of the LO between legitimate users and generate the LO locally at Bob’s side with an extra laser source, which can eliminate all of the above side-channel attacks effectively. In the protocol of self-referenced (SR) CV-QKD [23], the maximal transmission distance is positively correlated with the amplitude of the reference pulses. However, an extra excess noise proportional to the amplitude of the reference pulse will be generated due to the finite dynamics of Alice’s amplitude modulator [26]. This extra excess noise will limit the amplitude of the reference pulse and then greatly degrade the performance of the SR CV-QKD scheme, especially the maximal transmission distance. For example, for a more realistic value of the reference pulse amplitude of $V_{R}=20V_{A}$ ($V_{A}$ is the variance of the signal pulse), the maximal transmission distance is only around 5 km [23]. Therefore, it is of great practical significance to seek a solution to extend the maximal transmission distance when the reference pulse is weak.

Facing the issue of improving the secure transmission distance of the CV-QKD protocol, many approaches have been demonstrated to be useful. For example, the photon subtraction operation, a non-Gaussian operation that has been demonstrated theoretically and experimentally in CV-QKD [27,28,29,30,31,32], is an effective approach to enhance the transmission distance of CVQKD protocols significantly. Through the photon subtraction operation, the entanglement of Gaussian states can be enhanced; thus, the maximal transmission distance of CV-QKD protocols will be extended, and the noise tolerance of the states may be improved. However, the practical operation of photon subtraction will not only increase the physical complexity of the system, but also inevitably encounter the imperfections of devices, especially the single-photon detector. Fortunately, in the prepare-and-measurement (PM) scheme of CV-QKD with a coherent state, a real photon subtraction operation can be emulated by a non-Gaussian post-selection method, which can be deemed as a virtual photon subtraction [33]. This method not only has no need for complex physical operations, but also can emulate the ideal photon-subtraction operations. Therefore, the method of virtual photon subtraction is a superior way to improve the performance of CV-QKD protocols in practice, which has been demonstrated by many researches [33,34,35,36].

In this paper, we propose the scheme of SR CV-QKD with virtual photon subtraction. One advantage of using virtual photon subtraction is that it not only has no need for increasing the practical complexity of the original SR CV-QKD protocol, but also can emulate the ideal photon-subtraction operations. Another advantage is that it can extend the maximal transmission distance without increasing the intensity of the reference pulse and, thus, can effectively avoid the reference pulse’s leakage noise, which contributes to the finite dynamics of Alice’s amplitude modulator. Compared to the original SR CV-QKD protocol, our simulation results show that the maximal transmission distance can be extended considerably, especially for one-photon subtraction. Meanwhile, a lower electronic noise of Bob’s detector can bring about a longer extension of the maximal transmission distance. Moreover, our scheme can tolerate a larger excess noise than the original SR CV-QKD scheme, which implies the advantage of using virtual photon subtraction to increase the maximal tolerable excess noise for distant users. These results suggest that under existing technology, our modified scheme of the SR CV-QKD can make possible the SR CV-QKD from the laboratory for practical metropolitan area application.

This paper is organized as follows. In Section 2, we review the conventional Gaussian CV-QKD and the SR CV-QKD scheme. In Section 3, we first show the basic photon subtraction on a two-mode squeezed vacuum state, and then, we introduce our scheme of SR CV-QKD with virtual photon subtraction. In Section 4, we analyze the performance of our proposed scheme in the secure key rate and the maximal tolerable excess noise. Finally, we summarize this paper in Section 5.

## 2. The Conventional Gaussian and the SR CV-QKD Scheme

**Figure 1:**
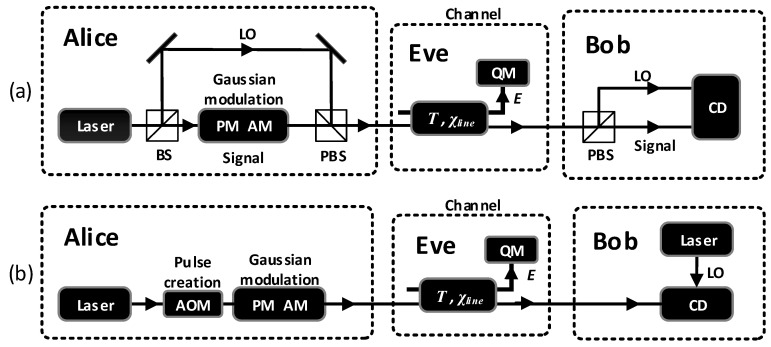
(**a**) The conventional Gaussian continuous-variable quantum key distribution (CV-QKD) scheme. The quantum signal and local oscillator (LO) are co-propagated from Alice to Bob. (**b**) The scheme of self-referenced (SR) CV-QKD. The quantum signals and reference pulses are co-transmitted through the same channel. At reception, the received pulses are measured in Bob’s own phase reference frame defined by the locally-generated LO. PM, phase modulator; AM, amplitude modulator; CD, coherent detection; QM, quantum memory; AOM, acousto-optical modulator; PBS, polarizing beam splitter; $\chi_{line}$, channel-added noise; T, channel transmission; E, Eve’s ancillae.

The conventional Gaussian CV-QKD scheme is illustrated in Figure 1a. Through the techniques of multiplexing in time and polarization, the quantum signals and the LO are co-transmitted from Alice to Bob in the quantum channel. Moreover, one can utilize the wavelength-division multiplexing technique to generate multiply-parallel quantum channels simultaneously, which are multiplexed and demultiplexed by the wavelength multiplexer and demultiplexer. At the receiver, Bob splits the quantum signals and the LO by the polarization controller and polarizing beam splitter. However, an eavesdropper can utilize the possible security loopholes of the intensity LO to perform side-channel attacks. Meanwhile, multiplexing and demultiplexing are knotty, as these are two kinds of signals that differ greatly in amplitude.

Different from the conventional Gaussian CV-QKD scheme, the SR CV-QKD scheme in [23] waives the transmission of the LO between legitimate users and operates essentially by employing a locally-generated LO, which effectively resists the possible side-channel attacks. The SR CV-QKD scheme could be generalized as shown in Figure 1b, and it contains two main steps:

Step 1: Alice prepares the Gaussian modulated coherent state $|q_{A}+ip_{A}\rangle$ as the quantum signal pulse and the other coherent state $|q_{A_{R}}+ip_{A_{R}}\rangle$ as the reference pulse. Then, she sends these coherent states to Bob without sending the LO. The two independent Gaussian random variables ($q_{A},p_{A}$) are both distributed as $N(0,V_{A})$, while the mean quadrature values of the reference pulse are fixed to $\left(q_{A_{R}},p_{A_{R}}\right)$ in Alice’s phase reference frame and are publicly known. The amplitude of the reference pulse $E_{R}$ ($E_{R}=\sqrt{p_{A_{R}}^{2}+q_{A_{R}}^{2}}$) may be several orders of amplitude larger than $\sqrt{V_{A}}$ and is much weaker than the amplitude of the LO.
Step 2: Bob performs a homodyne detection on the received signal pulse and a heterodyne detection on the reference pulse in his own reference frame defined by the locally-generated LO. He obtains $q_{B}$ or $p_{B}$ as one of the quadratures of the signal pulse and $q_{B_{R}}$ and $p_{B_{R}}$ as both of the quadratures of the reference pulse.

The reference pulse is used to estimate the phase deviation angle $\hat{\theta }$ between Alice’s and Bob’s reference frames. The $\hat{\theta }$ can be estimated by $\hat{\theta }=\theta +\phi$, where θ is the actual deviation angle and ϕ is the measurement error contributed by the quantum uncertainty. The covariance matrix between Alice and Bob can be written as [23]:

(1) $$\begin{array}{c} {\bar{\gamma }}_{AB}=\left(\begin{array}{cc} VI & C\bar{\cos\phi }\sigma_{Z}\\ C\bar{\cos\phi }\sigma_{Z} & T\eta (V+\chi )I \end{array}\right) \end{array}$$

with $C=\sqrt{T\eta (V^{2}-1)}$, where $I=\left(\begin{array}{cc} 1 & 0\\ 0 & 1 \end{array}\right)$ and $\sigma_{Z}=\left(\begin{array}{cc} 1 & 0\\ 0 & -1 \end{array}\right)$, *V* is the variance of Alice’s output state, χ is the channel noise, *T* is the channel transmission, η is the detector efficiency, $\bar{\cos\phi }=\int_{-\pi }^{\pi }d\phi P\left(\phi \right)cos\phi$ and P(ϕ) is the probability distribution of the random variable ϕ and is symmetric around ϕ=0.

According to the results in [23], the maximal transmission distance is positively correlated with the amplitude of the reference pulse. However, an extra excess noise proportional to the amplitude of the reference pulse will be generated due to the finite dynamics of Alice’s amplitude modulator [26]. Therefore, an arbitrary large amplitude of the reference pulse is not proper, and a more realistic value, such as $E_{R}^{2}=20V_{A}$, will be rational. Unfortunately, this realistic value will restrict the maximal transmission distance of the SR CV-QKD protocol to a fairly low level, as illustrated in Section 4 later on. This issue will hinder the practical application of the SR CV-QKD scheme.

## 3. SR CV-QKD with Virtual Photon Subtraction

**Figure 2:**
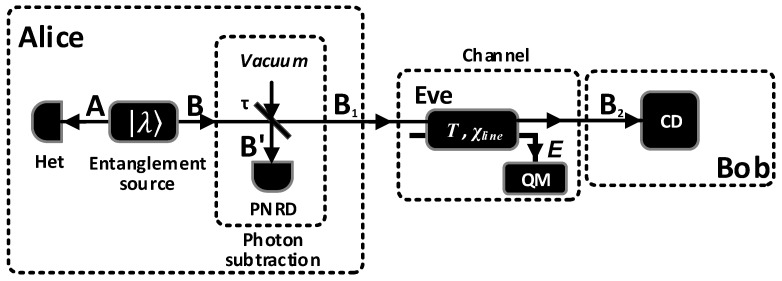
Schematic of EB CV-QKD with photon subtraction. PNRD: photon number resolving detector; Het: heterodyne detection; CD: coherent detection; QM: quantum memory.

Photon subtraction can improve the entanglement of the two-mode squeezed vacuum (TMSV) state and hence enhance the performance of the system. In order to make the description of our scheme self-contained, we first start with the basics of photon subtraction on a TMSV state. Figure 2 describes the entire steps of the EB CV-QKD scheme with photon subtraction. An entanglement source |λ〉 is used to produce the TMSV state and $|\lambda \rangle =\sqrt{1-\lambda^{2}}\sum_{n=0}^{\infty }\lambda^{n}|n,n\rangle$. Then, Alice performs heterodyne detection on mode *A* and sends mode *B* to a beam splitter (BS) with transmittance τ. The mode *B* is split into modes: $B^{\prime }$ and $B_{1}$. The modes *A*, $B^{\prime }$, $B_{1}$ form a tripartite state $\rho_{AB^{\prime }B_{1}}$,

(2) $$\rho_{AB^{\prime }B_{1}}=U_{BS}[|\lambda \rangle \langle \lambda |⊗|0\rangle \langle 0|]U_{BS}^{†}.$$

The photon number resolving detector (PNRD) is used to perform the positive operator-valued measure (POVM) $\left\{{\hat{\Pi }}_{0},{\hat{\Pi }}_{1}\right\}$ on mode $B^{\prime }$ [37]. Only when the POVM elements ${\hat{\Pi }}_{1}$ click, the mode *A* and $B_{1}$ can be kept. The kept state is given by:

(3) $$\rho_{AB_{1}}^{{\hat{\Pi }}_{1}}=\frac{tr_{B^{\prime }}\left({\hat{\Pi }}_{1}\rho_{AB^{\prime }B_{1}}\right)}{tr_{AB^{\prime }B_{1}}\left({\hat{\Pi }}_{1}\rho_{AB^{\prime }B_{1}}\right)},$$

where $tr_{x}\left(\cdot \right)$ is the partial trace of the multimode quantum state and $P^{{\hat{\Pi }}_{1}}=tr_{AB^{\prime }B_{1}}\left({\hat{\Pi }}_{1}\rho_{AB^{\prime }B_{1}}\right)$ denotes the success probability of subtracting *k* photons.

**Figure 3:**
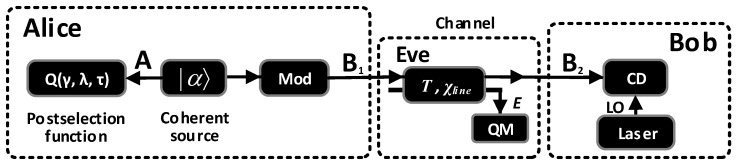
Schematic of PM SR CV-QKD with virtual photon subtraction. QM: quantum memory; CD: coherent detection; Mod: Gaussian modulator.

However, the straightforward application of the above photon subtraction to the SR CV-QKD is not a desirable method, in which the reference pulse will also pass through the BS and the hardware requirement will be enhanced. Fortunately, the EB CV-QKD scheme with photon subtraction can be equivalent to the PM CV-QKD scheme with virtual photon subtraction via non-Gaussian post-selection [33]. In the post-selection step, Alice uses a post-selection filter function Q(·), or acceptance probability, to decide which data will be accepted. The post-selection step is carried out after Bob has performed coherent detection, which means it will not change the Gaussian state $\rho_{AB_{2}}^{G}$ and the Gaussian process G. The mode $B_{2}$ is the received mode at Bob’s side. Therefore, we propose the scheme of SR CV-QKD with virtual photon subtraction, which can be realized via non-Gaussian post-selection. The schematic diagram of our scheme is described in Figure 3, where $\alpha =\sqrt{2\tau }\lambda \gamma /2$, and γ is the measurement result of mode *A* in the EB scheme, i.e., $\gamma =x_{A}+ip_{A}$. The modulation variance of $x_{A}$ and $p_{A}$ is $\tilde{V}=\left(V+1\right)/2$, where $V=\left(1+\lambda^{2}\right)/\left(1-\lambda^{2}\right)$ is the variance of the TMVS state in the EB scheme. Hence, according to the derived results in [33], the covariance matrix ${\bar{\gamma }}_{AB_{2}}^{G}$ of the Gaussian state $\rho_{AB_{2}}^{G}$ for subtracting *k* photons can rewrite Equation (1) as:

(4) $$\begin{array}{c} {\bar{\gamma }}_{AB_{2}}^{G}=\left(\begin{array}{cc} V_{A}I & \bar{C}\sigma_{Z}\\ \bar{C}\sigma_{Z} & V_{B}I \end{array}\right) \end{array}$$

with:

(5) $$\begin{array}{ccc} V_{A} & = & 2V_{k}-1, \end{array}$$

(6) $$\begin{array}{ccc} V_{B} & = & T_{e}\left(2\tau \lambda^{2}V_{k}+1+\chi \right), \end{array}$$

(7) $$\begin{array}{ccc} \bar{C} & = & 2\sqrt{T_{e}\tau }\lambda V_{k}\bar{\cos\phi }, \end{array}$$

(8) $$\begin{array}{ccc} \chi & = & \frac{\left(1-T_{e}\right)}{T_{e}}+\frac{\varepsilon_{el}}{T_{e}}+\varepsilon_{c}, \end{array}$$

(9) $$\begin{array}{ccc} V_{k} & = & \frac{k+1}{1-\tau \lambda^{2}}, \end{array}$$

where $\varepsilon_{el}$ is the electronic noise of the Bob’s detector, $\varepsilon_{c}$ is the channel excess noise and $T_{e}=T\eta$.

## 4. Performance Analysis

Usually, the secret key rate of the TMSV state is no less than the secret key rate of the equivalent Gaussian state, which shares an identical covariance matrix due to the extremality of Gaussian state [38,39,40]. Hence, we will use ${\bar{\gamma }}_{AB_{2}}^{G}$ to derive the lower bound of the secret key rate. Besides, the acceptance probability for each of the data in the post-selection step should also be taken into account. This probability is equivalent to the success probability of Alice’s POVM measurement $P^{{\hat{\Pi }}_{1}}$ and can be treated as a scaling factor.

### 4.1. Individual Attacks

The lower bound of the secret key rate of our scheme against individual attack for reverse reconciliation is:

(10) $$\begin{array}{c} K_{min}^{ind}=P^{{\hat{\Pi }}_{1}}\left(\beta I_{AB}^{G}-I_{EB}\right), \end{array}$$

where $P^{{\hat{\Pi }}_{1}}=\frac{1-\lambda^{2}}{1-\tau \lambda^{2}}{\left[\frac{\lambda^{2}\left(1-\tau \right)}{1-\tau \lambda^{2}}\right]}^{k}$ [33], $I_{AB}^{G}$ is the mutual information between Alice’s and Bob’s measurements, $I_{EB}$ is mutual information between Eve’s and Bob’s measurements and β is the reconciliation efficiency.

From the covariance matrix in Equation (4) and the derived results in [23], the mutual information between Alice’s and Bob’s measurements $I_{AB}^{G}$ can be written as:

(11) $$I_{AB}^{G}=\frac{1}{2}\log_{2}\left(\frac{V^{\prime }}{V_{A|B}}\right)$$

with $V^{\prime }=\left(V_{A}+1\right)/2$ and $V_{A|B}=V^{\prime }-{\bar{C}}^{2}/2V_{B}$. Through the relationship:

(12) $$\begin{array}{c} 1-{\bar{cos\phi }}^{2}=V_{\hat{\theta }}=\frac{\chi +1}{V_{R}}+\frac{\delta_{R}}{T\eta V_{R}}, \end{array}$$

we can get:

(13) $$\begin{array}{c} {\bar{C}}^{2}=4T_{e}\tau \lambda^{2}V_{k}^{2}{\bar{cos\phi }}^{2}=4T_{e}\tau \lambda^{2}V_{k}^{2}\left(1-V_{\hat{\theta }}\right), \end{array}$$

where $V_{R}=E_{R}^{2}$, $\delta_{R}=1$ for single-reference-pulse mode and $V_{\hat{\theta }}$ is the variance of the estimated deviation angle $\hat{\theta }$. The upper bound of mutual information between Eve’s and Bob’s measurements can be given by:

(14) $$\begin{array}{c} I_{EB}=\frac{1}{2}\log_{2}\left(\frac{V_{B}}{V_{B|E}}\right)=\frac{1}{2}\log_{2}\left(V_{B}V_{B|A}\right) \end{array}$$

with $V_{B|A}=V_{B}-{\bar{C}}^{2}/V_{A}$.

### 4.2. Collective Attacks

The asymptotic secret key rate against collective attacks for reverse reconciliation can be given by:

(15) $$\begin{array}{c} K_{min}^{col}=P^{{\hat{\Pi }}_{1}}\left(\beta I_{AB}^{G}-\chi_{BE}^{G}\right), \end{array}$$

where $I_{AB}^{G}$ is given by Equation (11) and $\chi_{BE}^{G}$ is the maximal stolen information. The maximal stolen information $\chi_{BE}^{G}$ can be written as:

(16) $$\chi_{BE}^{G}=G\left(\frac{\lambda_{1}-1}{2}\right)+G\left(\frac{\lambda_{2}-1}{2}\right)-G\left(\frac{\lambda_{3}-1}{2}\right),$$

where $G\left(x\right)=\left(x+1\right)\log_{2}\left(x+1\right)-x\log_{2}\left(x\right)$ is the von Neumann entropy of a thermal state. The eigenvalues $\lambda_{1}$ and $\lambda_{2}$ are obtained from:

(17) $$\begin{array}{c} \lambda_{1,2}^{2}=\frac{1}{2}\left(\Delta \pm \sqrt{\Delta^{2}-4D^{2}}\right) \end{array}$$

with $\Delta =V_{A}^{2}+V_{B}^{2}-2{\bar{C}}^{2}$ and $D=V_{A}V_{B}-{\bar{C}}^{2}$. The square of symplectic eigenvalue $\lambda_{3}$ reads:

(18) $$\begin{array}{c} \lambda_{3}^{2}=V_{A}\left(V_{A}-\frac{{\bar{C}}^{2}}{V_{B}}\right). \end{array}$$

**Figure 4:**
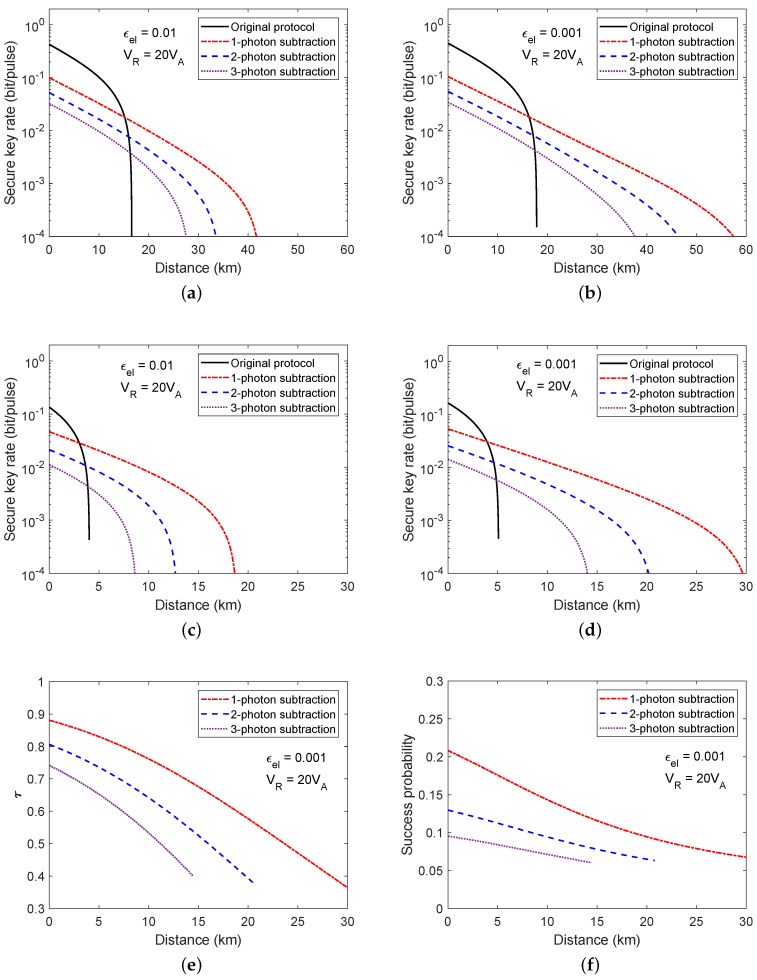
The simulation results against individual and collective attacks. (**a**,**b**) give the maximum secure key rate as a function of the transmission distance against individual attacks, when changing the transmittance τ of Alice’s beam splitter (BS); (**c**,**d**) give the maximum secure key rate against collective attacks; (**e**) shows the optimal τ corresponding to (d); (**f**) is the success probability of subtracting kphotons at each transmission distance corresponding to (d). The black solid lines show the original SR-CV-QKD protocol without photon subtraction. Other lines represent one-photon subtraction (red dashed-dotted lines), two-photon subtraction (blue dashed lines), three-photon subtraction (violet dotted lines).

In what follows, we will assume that $V_{A}=40$, β=0.95, $\varepsilon_{c}=0.01$, η=0.719 and α=0.2 dB/km [23]. All the variances in this paper are in shot-noise units. Figure 4 shows our simulation results against individual and collective attacks. Figure 4a–d gives the maximum secure key rate at each transmission distance for all possible τ of Alice’s BS. Note, due to the excess noise contributed by the leakage of reference pulses, here, $V_{R}$ is set to a more realistic value of 20$V_{A}$, and thus, we neglect this excess noise (about $8\times 10^{-4}$ when the dynamics of Alice’s amplitude modulator is 60 dB) [26]. The figures show a considerable maximal transmission distance improvement when the photon subtraction operation is applied in the SR CV-QKD scheme, especially in the case of subtracting one photon. Furthermore, we find that our scheme of SR CV-QKD with virtual photon subtraction is sensible with the detector electronic noise. A lower electronic noise can result in a larger maximal transmission distance, as shown in Figure 4b,d. We note that the electronic noise of 0.001 is achievable, which is demonstrated in [41]. However, the secure key rate is worse than the original protocol in the short distance region. The main reason for this phenomenon is that the limited acceptance probability degrades the final key rate. τ is a key parameter, which should be determined in advance. Figure 4e shows the optimal τ at each distance for the maximum secure key rate in Figure 4d. The optimal τ decreases along with the increasing of the transmission distance, which implies a accurate estimation of τ is required for each distance. Figure 4f represents the success probability of subtracting *k* (*k* = 1, 2, 3) photons at each distance for the maximum secure key rate in Figure 4d. Although the success probability will be larger in the region of large τ, a large success probability does not mean a large secure key rate, especially when the transmission distance becomes longer. This is because τ not only impacts the success probability, but also the entire key generation. We did not draw the optimal τ and the success probability of subtracting *k* photons at each distance for the maximum secure key rate in Figure 4c, as their results are similar to the case when the electronic noise is 0.001.

**Figure 5:**
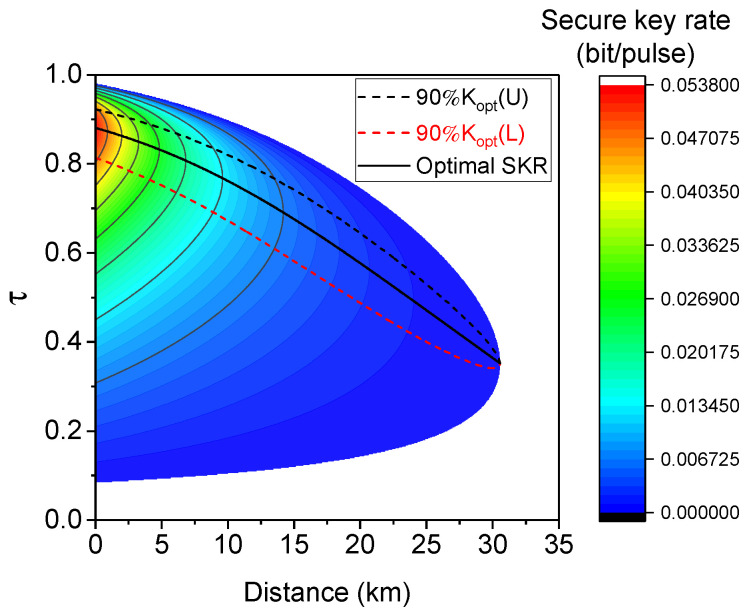
The secure key rate as a function of transmission distance and τ of Alice’s BS, when the electronic noise is 0.001. The black solid line is the optimal τ, while the secure key rate reaches its maximal value at each distance. The black (red) dashed line is the upper (lower) bound of τ, when its secure key rate is 90% of its maximum at that distance.

From a practical point of view, if the secure key rate varies rapidly with τ around its optimal value, the accurate estimation of the optimal τ will need complicated implementations. Fortunately, around the optimal value of τ, the secure key rate varies slowly with the change of τ at each distance, as shown in Figure 5. Particularly, between the upper bound (black dashed line) and lower bound (red dashed line) of τ at a specific distance, the secure key rate can maintain more than 90% of its optimal value ($K_{opt}$).

**Figure 6:**
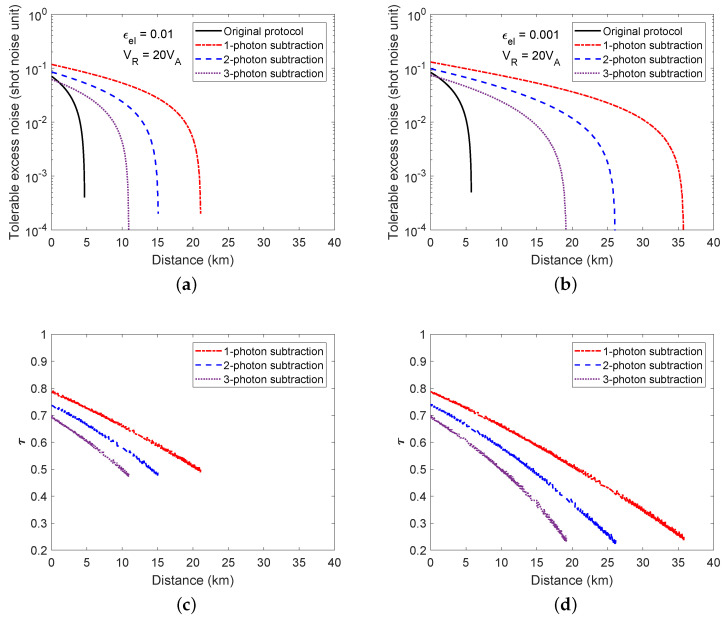
The maximal tolerable excess noise and its corresponding value of τ at each distance. (**a**,**b**) are the maximal tolerable excess noise at each distance for all possible τ when electronic noise is equal to 0.01 and 0.001; (**c**,**d**) are the optimal τ for the maximal tolerable excess noise corresponding to (a,b).

Another aspect of our scheme is the tolerable excess noise. As shown in Figure 6a,b, we depict the relationship between the maximal tolerable excess noise and the transmission distance for different electronic noise and all possible τ. The original scheme is outperformed by the protocol of using photon subtraction at all transmission distance ranges, which implies the advantage of using photon subtraction, which increases the maximal tolerable excess noise for distant users. Moreover, if the channel is less noisy, for example, $\varepsilon_{c}\approx 0.005$, the one photon subtraction can expand the maximal transmission distance to 20 km for $\varepsilon_{el}=0.01$ and 33 km for $\varepsilon_{el}=0.001$. As the tolerable excess noise is not affected by the acceptance probability, the optimal τ for the maximal tolerable excess noise at each distance is different from that of the one for the maximum secure key rate, as shown in Figure 6c,d.

Actually, many protocols have investigated the CV-QKD with virtual photon subtraction. All of them can significantly improve the maximal transmission distance of the CV-QKD protocols. In [33], the method of virtual photon subtraction was firstly used in the conventional one-way GMCS CV-QKD scheme, the LO of which is co-transmitted with the quantum signal. The maximal transmission distance can be extended from 90–220 km (144% improvement). The protocol of two-way GMCS CV-QKD with virtual photon subtraction was investigated in [35]. The maximal transmission distance can be extended from 85–310 km (266% improvement). In [34], the four-state CV-QKD protocol combined with virtual photon subtraction can extend the maximal transmission distance from 140–330 km (136% improvement). For the protocol of measurement-device-independent CV-QKD with virtual photon subtraction, the maximal transmission distance can be extended from 42–68 km (62% improvement) [36]. In our scheme of SR CV-QKD with virtual photon subtraction, we also obtained a considerable extension of the maximum transmission distance when the detector electronic noise was 0.001. The maximum transmission distance increased from 18–58 km (222% improvement) under individual attack and from 5–30 km (500% improvement) under collective attacks, which makes possible the application of the SR CV-QKD from the laboratory to an actual metropolitan area. If we increase the amplitude of the reference pulse appropriately and control the reference pulse’s leakage noise in a certain range, the maximum transmission distance can be extended further. For example, if $V_{R}=50V_{A}$ and the detector electronic noise is equal to 0.001, the maximal transmission distance can be extended from 15–40 km. In practice, the imperfection of the detector will constrain the performance of the CV-QKD protocol. Therefore, any imperfection of the detector should be taken into account, while this was not considered in [34,36,37].

## 5. Conclusions

In this paper, we proposed the scheme of SR CV-QKD with virtual photon subtraction. It not only has no need to increase the physical complexity of the original SR CV-QKD system, but also can extend the maximal transmission distance without increasing the intensity of the reference pulse. Performance analysis results show that a considerable extension of maximal transmission distance can be obtained, especially for one-photon subtraction. Meanwhile, the scheme of SR CV-QKD with virtual photon subtraction is sensible with the detector’s electronic noise. A longer maximal transmission distance can be obtained when the electronic noise is lower. Furthermore, it is more tolerable against excess noise for our scheme compared to the original protocol, which implies the advantage of using virtual photon subtraction to increase the maximal tolerable excess noise for distant users. These results suggest that under existing technology, our modified scheme of the SR CV-QKD can make possible the SR CV-QKD from the laboratory to practical metropolitan area application. However, we note that the gap between practical implementations and the theoretical analysis here should be taken into account. Any imperfection factors in the practical experiment should introduce corresponding parameters. This issue is not included in the scope of the present analysis, and deserves further study.
